# Efficacy Evaluation of a Multifunctional Cosmetic Formulation: The Benefits of a Combination of Active Antioxidant Substances

**DOI:** 10.3390/molecules191118268

**Published:** 2014-11-10

**Authors:** Mirela D. Gianeti, Patrícia M. B. G. Maia Campos

**Affiliations:** Faculty of Pharmaceutical Sciences of Ribeirao Preto, University of Sao Paulo, Av do Cafe s/n, Monte Alegre, Ribeirao Preto, SP 14040-903, Brazil

**Keywords:** multifunctional cosmetics, image analysis, clinical efficacy, skin aging, antioxidants

## Abstract

This study presents the association of active antioxidants substances in a multifunctional cosmetic formulation with established efficacy against signs of aging. A multifunctional cosmetic formulation containing an association of UV filters and antioxidant substances (liposoluble vitamins A, C and E, *Ginkgo biloba* and *Phorphyra umbilicalis* extracts) was evaluated. This formulation was submitted to a clinical efficacy study using biophysics techniques and skin images analysis (digital photography imaging systems, 20 MHz ultrasound, and reflectance confocal microscopy). The volunteers applied the formulation containing the UV filters and antioxidant substances during the day and the formulation with antioxidant substances and without the UV filters at night, for 90 days. The formulation increased the hydration and protected the skin barrier function after a single application. At the long term assessment the formulation provided an improvement in skin barrier function and skin hydration to the deeper layers of the epidermis, leading to an improvement in skin appearance by reducing wrinkles and skin roughness. The multifunctional cosmetic formulation studied can be suggested to preventing signs of aging and improving skin conditions. In addition, this study presents the benefits of associating different active antioxidants substances in a single cosmetic formulation to prevent skin aging.

## 1. Introduction

Oxidative stress plays an important role in skin aging [1], especially extrinsic skin aging. This phenomenon is caused by reactive oxygen species (ROS), while prolonged exposure to UV light diminishes the antioxidant capacity of the skin [2,3].

The search for better cosmetic products has prompted the development of multifunctional cosmetic formulations that associate different active substances. In particular, combining sunscreens with vitamins and vegetable extracts culminates in antioxidant and protective effects that improve skin appearance and prevent the damage caused by UV radiation and oxidative stress [4].

Many research groups, including ours, have demonstrated that cosmetic formulations combining inorganic (TiO_2_) and organic (ethylhexyl methoxycinnamate, benzophenone, and octocrylene) UV filters with vitamin derivatives (retinyl palmitate, ascorbyl tetraisopalmitate, and tocopheryl acetate) and botanical extracts (*Ginkgo biloba* and *Phorphyra umbilicalis*) display enhanced antioxidant activity and increased protection against UV radiation [4,5,6,7,8].

The application of active antioxidant substances in cosmetic formulations stems from their ability to protect the skin against oxidative damage by UV radiation and aging. However, little is known about the effect of these antioxidants on improving skin conditions and preventing signs of age.

This study presents the association of active antioxidants substances in a multifunctional cosmetic formulation with established efficacy against signs of age, assessed by biophysical techniques and image analysis.

In this context, the association of ultraviolet (UV) filters with antioxidants such as vitamins which can offer unique benefits in protecting the skin, acting synergistically against free radicals produced by exposure to UV radiation, which leads to prevention of biological damage and even a possible reversal of skin aging [9] must be emphasized. In addition, some botanical extracts have also attracted great interest for use in anti-aging cosmetic products with the purpose of reducing free radical damage due to the presence of bioflavonoids in their composition [10,11,12,13].

Among these extracts the use *Ginkgo biloba* extract in anti-aging cosmetic products has been proposed due to its composition rich in flavonoids (rutin, quercetin, kaempferol), biflavones and terpenoids related to its properties antioxidant and anti-inflammatory [8]. Studies have shown that *Ginkgo biloba* extract protected the skin of hairless mice against skin barrier function damage and against erythema induced by ultraviolet radiation [6]. Consequently, a large number of products for skin care have included this ingredient in its composition [8,9]. The association of *Ginkgo biloba* extract with vitamins (vitamins A, C and E) in cosmetic formulations also showed antioxidant activity *in vitro* and protected the skin against damage induced by UV radiation [6].

Apart from vegetable extracts with antioxidant activity, botanical extracts rich in amino acids, mycosporine-like amino acids—MAA, have been proposed by the scientific community as presenting potential protection against UV radiation [14,15,16]. These substances when used in combination with synthetic UV filters may provide greater protection of the skin and increase sun protection factor (SPF). Moreover, studies in humans have shown that after four weeks using a formulation containing MAA-rich extracts skin firmness and smoothness were improved, and a reduction of wrinkles was also observed [17]. Among these extracts the one obtained from red sea algae *Porphyra umbilicalis*, which has been proposed because of the high concentration of active substances responsible for the protective effect against solar radiation [5,15] could be highlighted.

Vitamins A, C and E, and their derivatives, have been widely used in cosmetic products alone or in combination with other active ingredients due to their moisturizing, antioxidant and anti-aging properties [7,10,18,19].

The ester forms of vitamins A, C and E (retinyl palmitate, ascorbyl tetraisopalmitate and tocopheryl acetate, respectively) have been used because of their greater stability [18,20,21]. Therefore, studies have been conducted to demonstrate the effectiveness of these derivatives soluble vitamins on human skin.

Moreover, considering that several endogenous antioxidants suffer sudden reduction with age and also after exposure to UV radiation [22], the association of antioxidants and sunscreens has been widely used due to their synergistic effects in protecting the skin against free radicals produced by exposure to ultraviolet radiation, which leads to prevention of damage to and even possible reversal of photoaging [23,24].

In this context, we suggest the association of fat-soluble derivatives of vitamins A, C and E, botanical extracts such as *Ginkgo biloba* and *Porphyra umbilicalis* in the development of multifunctional formulation with the proposal to prevent damage to the skin caused by sun exposure and improve the appearance of aged skin. Thus, the aim of this study was to evaluate the clinical efficacy of a multifunctional cosmetic formulation containing sunscreens, antioxidant liposoluble vitamins and *Ginkgo biloba* and *Phorphyra umbilicalis* extracts.

## 2. Results and Discussion

**Figure 1:**
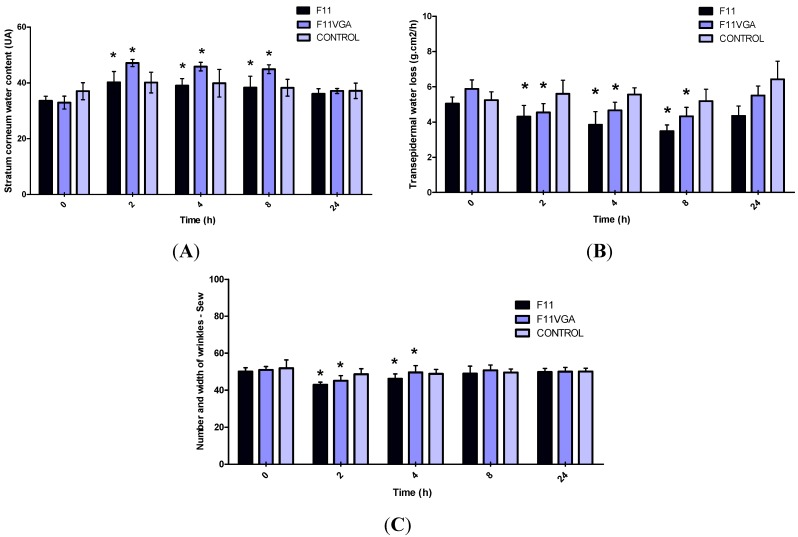
Mean and confidence interval 95% (CI95%) achieved after a single application of studied formulations for the parameters: stratum corneum water content (**A**), transepidermal water loss (**B**) and number and width of wrinkles—Sew (**C**), evaluated on the forearms of the volunteers (N = 45 volunteers).

An increase in the water content of the stratum corneum and protection of the skin barrier function was observed 2 h after a single application of the studied formulation which was maintained until 8 h after the first application (Figure 1). These effects were not observed 24 h after a single application of the studied formulation.

Moreover, a decrease in Sew, a microrelief parameter related to the number and width of wrinkles, was observed 2 h and 4 h after application (Figure 1C).

Changes in cutaneous microrelief parameters observed after a single application of the formulations are related to a moisturizing effect, since the hydration may provide an immediate improvement in skin appearance mostly with the reduction of width and number of wrinkles [4,25].

**Figure 2:**
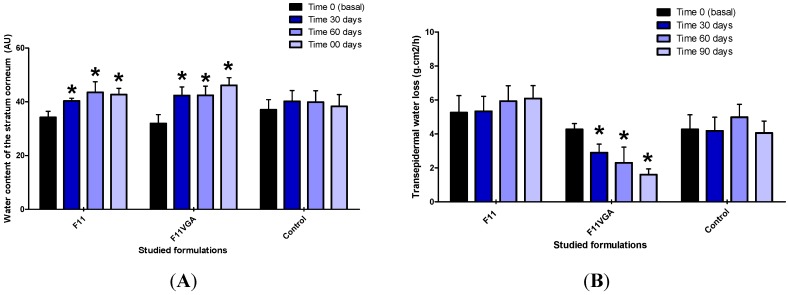

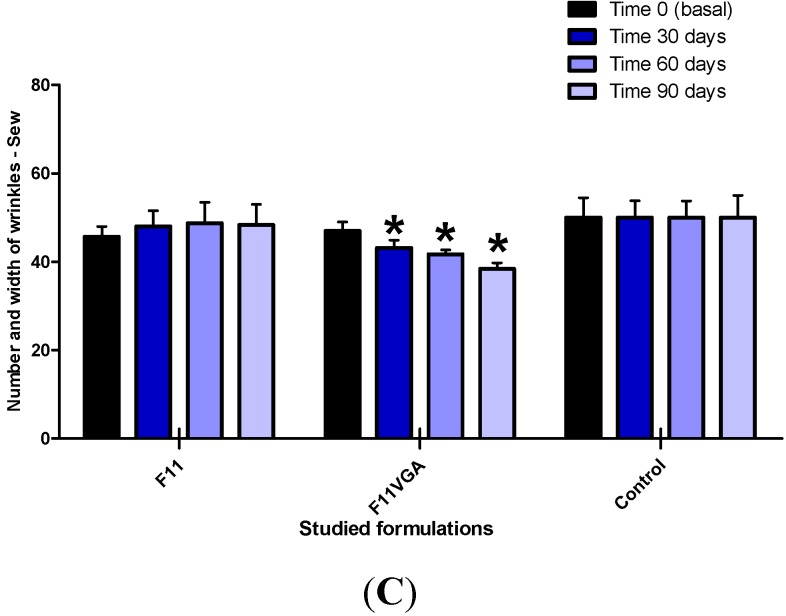
Mean and confidence interval 95% (CI95%) achieved after 30, 60 and 90 days of daily application of studied formulations for the parameters: water content of the stratum corneum (**A**), transepidermal water loss (**B**) and number and width of wrinkles—Sew (**C**), evaluated on the forearms of the volunteers (N = 45 volunteers).

At the long term assessment study, the multifunctional proposed formulation containing active antioxidant substances provided an improvement in skin barrier function and hydration until the deeper layers of the epidermis, shown by an increase in the stratum corneum water content as well as a decrease in TEWL and Sew parameters (Figure 2) [12].

**Figure 3:**
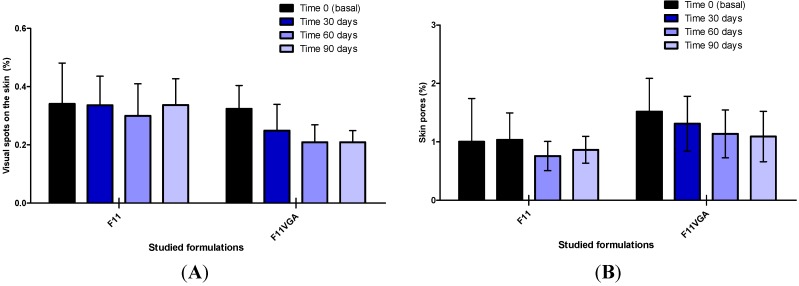
Mean percentage and confidence interval 95% (CI95%) of visual spots (**A**) and skin pores (**B**) achieved on the face of volunteers after 30, 60 and 90 days of daily application of studied formulations (N = 45 volunteers).

Despite the moisturizing effect, the improvement on transepidermal water loss may be related with the protective effect of active antioxidant substances against the damages caused by sun exposure. In relation to the analysis of skin made using digital photography imaging systems, a non-significant decrease in the percentage of spots and the percentage of pores on the skin was observed with the use of the multifunctional formulation (Figure 3), suggesting that the proposed combination of active substances can enhance the appearance of the skin, making it more homogeneous.

*: Statistically different from baseline values.

*: Statistically different from baseline.

Once oxidative stress has been suggested to play a role in ultraviolet (UVA) damage mediated melanogenesis and reduction on skin elasticity, through promoting oxidative stress, which occurs as the result of increased formation of oxidants and/or reactive nitrogen species (RNS) including nitric oxide (NO), this effect of improvement on skin color and appearance of pores could be related to the active antioxidant substances associated in the multifunctional studied formulation [26,27,28,29,30].

**Figure 4:**
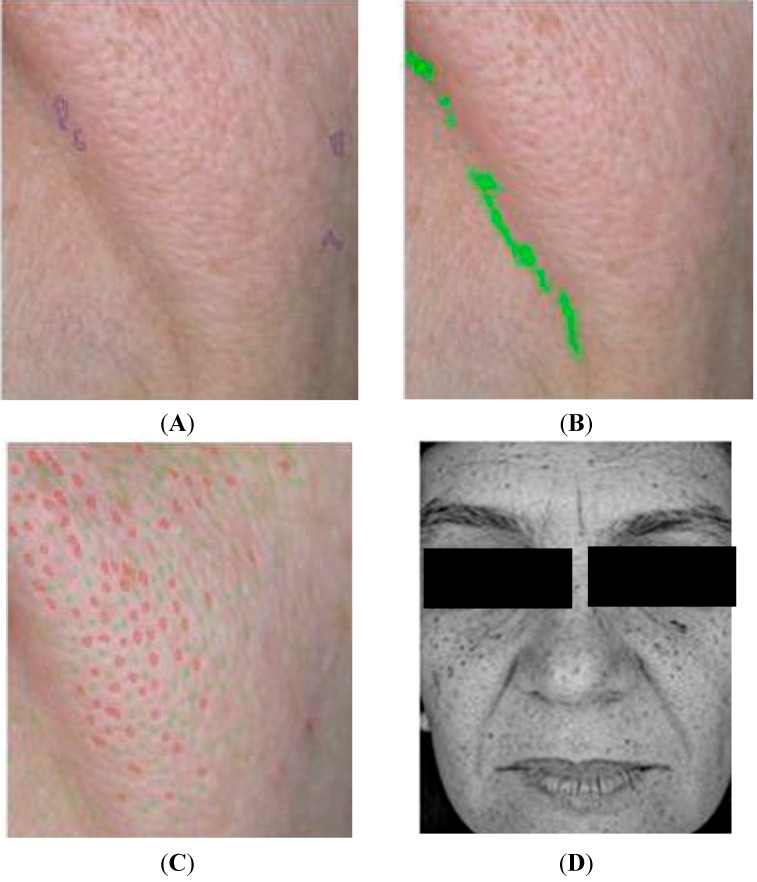
Illustrative images obtained with the digital photography imaging system: visible spots (**A**); wrinkles (**B**); pores (**C**); and spots visible only under UV light (**D**).

Figure 4 shows the parameters evaluated with digital photography imaging system. No statistically significant differences were observed with the use of the studied formulations regarding the percentage of winkles and spots observed with the UV light.

Twenty MHz ultrasound is a safe, non-invasive technique for the evaluation of the skin changes in the dermis; e.g., thickness and echogenicity [31,32]. This equipment is very important and has been used in the diagnosis of skin cancer, since it aids surgical planning in a decisive way [31].

**Figure 5:**
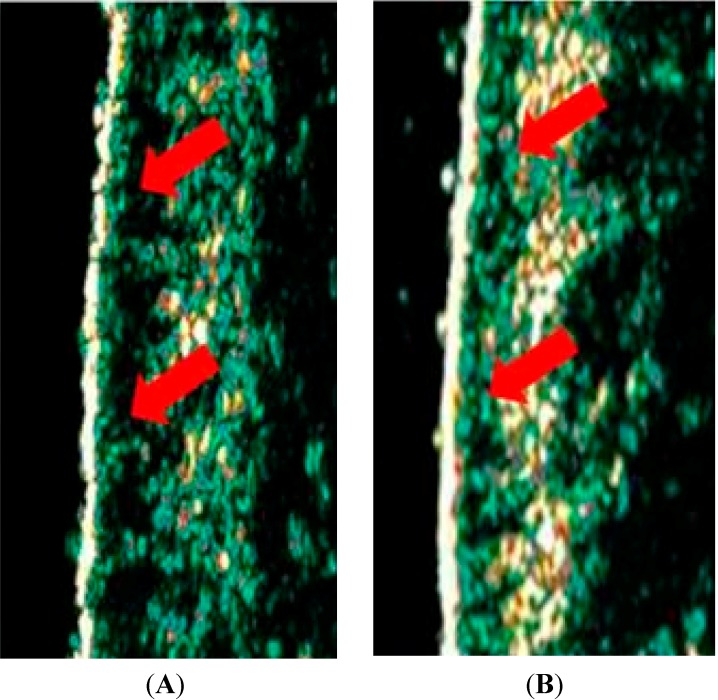
Illustrative images obtained on the face with the 20 MHz ultrasound of an elderly volunteer (**A**) and of a younger volunteer (**B**), highlighted hypoechogenic bands.

No significant differences were observed in the dermis echogenicity after application of the formulations when evaluated by 20 MHz ultrasound. However signs of photoaging were observed in elderly volunteers presented by a hypoechogenic bands (dark), which usually appear in the epidermal-dermal junction (Figure 5) and have been correlated with photoaging severity (Figure 5) [33,34], showing the importance of using UV filters and antioxidants in cosmetic formulations. This device represents an effective method to evaluate changes in human skin, therefore suggested to evaluate the efficacy of anti-aging formulations [33,34].

**Figure 6:**
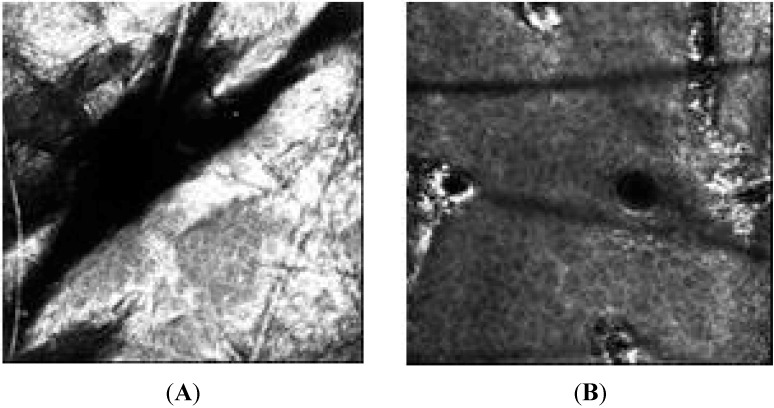

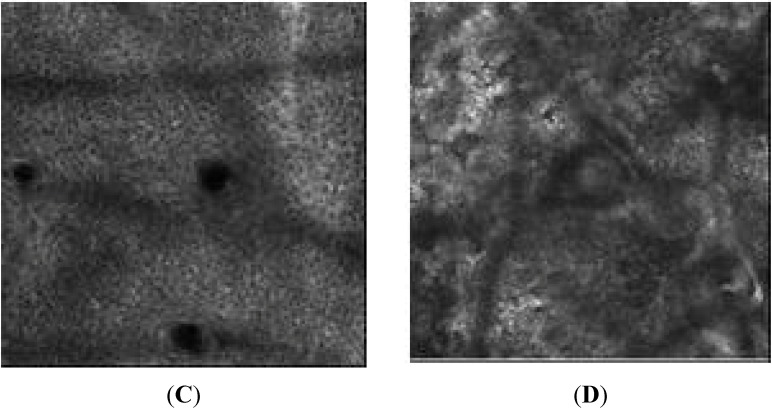
Images obtained by confocal laser microscopy of the different skin layers: (**A**) stratum corneum (**B**) granular layer, spinous layer (**C**) and basal layer (**D**).

In addition to traditional techniques of anti-aging efficacy evaluation, in the present study confocal laser microscopy has been proposed to assess the efficacy of studied formulation through the evaluation of the epidermal layers as presented by representatives in Figure 6. It should be noted that few studies have used this innovative technology to evaluate the efficacy of cosmetic and also, this was the first time in Brazil.

**Figure 7:**
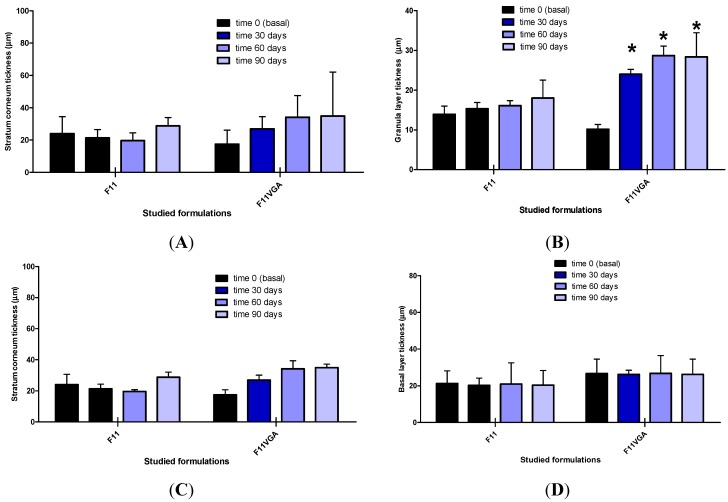
Average and confidence interval 95% (CI95%) thickness of stratum corneum (**A**); spinous layer (**B**); granular layer (**C**) and basal layer (**D**) before (baseline) and after 30, 60 and 90 days of application of the studied formulations in the face of the volunteers (N = 45 volunteers).

The average thickness of the different layers of the epidermis is presented in Figure 7.

*: Statistically different from baseline.

Among the numerous parameters that this technique allows us to evaluate, we highlight that the multifunctional formulation statistically increased the thickness of the granular layer (Figure 7B), and a non-significant increase in the stratum corneum and spinous layer, was also observed, which was not observed with the application of control (F11) (Figure 7A,C,D).

This effect could be linked to the moisturizing effect of the active substances studied, showing that the multifunctional formulation provided an increased water content in surface (stratum corneum and reduction on transepidermal water loss) and in the deeper layers of the epidermis [11,25], which enhanced the appearance of the skin with reduction of wrinkles [35].

## 3. Methods

### Formulation Studied

Two different formulations based on polyacrylamide, C13–14 isoparaffin & Laureth-7; polyglyceryl-10 pentaestearate and sodium estearoyl lactate; silicone microemulsion; propyleneglycol; glycerin; phenoxyethanol and parabens; butylhydroxytoluene—BHT; disodium EDTA and water were developed. To this vehicle was added (F11VGA—multifunctional formulation) or not (F11—vehicle) with the active substances under study (liposoluble vitamins A, C and E, *Ginkgo biloba* and *Phorphyra umbilicalis* extracts).

Vitamin A, C and E derivatives working in many biologic systems for protection against free radicals [7,8,9] predominantly protect lipid structures in skin, including membranes. Consequently, vitamins A, C and E can be considered as natural antioxidants that protect the skin from oxidative stress generated by sunlight and other environment factors [7]. These vitamin derivatives are capable of inhibiting free radical production by 10%–20% [7].

The *Phorphyra umbilicalis* extract (from Mibelle AG Biochemistry—Buchs, Switzerland) is a methanol extract rich in proteins, vitamins, minerals and mainly in MAA’s porphyra-334 and shinorine concentrated in liposomes (total concentration of 1.4% of phorphyra-334 and shinorine). The compounds of *Phorphyra umbilicalis* can reduce lipid peroxidation by 37% [17].

The *Ginkgo biloba* extract (from Alban Muller International—Vincennes, France) is a glycolic extract standardized in quercetin (its chemical marker) obtained from the leaves of the plant. Plant polyphenolic compounds like quercetin present in *Ginkgo biloba* extract have a broad biological profile, which is related to their antioxidant/free-radical scavenging capability and thus to their efficacious protective effect under oxidative stress conditions, including UV-induced damage. Quercetin have been shown to interfere not only with the propagation reaction in the lipoperoxidative chain, but also with the formation of free radicals, either by chelating the transition metal or by inhibiting the enzymes involved in the initiation reaction [6,7]. The concentration of these active substances is presented in Table 1.

Also, the formulations were added or not with a mixture of UV filters. The vehicle and the multifunctional formulation containing the UV filters were developed to be applied during the day, as well the formulations without the UV filters were developed to be applied at night.

**Table 1:** Concentration of the active substances used in the development of the multifunctional cosmetic formulation.

Active Substance	Concentration (%)
*Ginkgo biloba* extract	1.5
*Phorphyra umbilicalis* extract	5.0
Vitamin A (Retinyl Palmitate)	0.4
Vitamin C (Ascorbyl Tetraisopalmitate)	0.06
Vitamin E (Tocopheryl Acetate)	0.4

In addition, once the studied formulations presented an association of active substances, they were extensively evaluate regarding their chemical and physical stability before being applied on human skin for efficacy evaluation [24,25] and, because of their composition of inorganic UV filters their photocatalytic activity was also evaluated [36]. No signs of instability were observed during this study and it was also noted that the formulation F11VGA undergoes significantly slower oxidation than F11, which is probably a consequence of the combined antioxidant activity of the active substances introduced in the formulation.

It is also important to mention that, although TiO_2_ used as inorganic UV filter in the photoprotective formulations has a potential photocatalytic effect that may indirectly to the sun exposure induce damage to the skin DNA [37], the coated TiO_2_ used in the development of the multifunctional cosmetic formulation under study has not presented this effect. In addition, considering the statement above and that the multifunctional cosmetic formulation were extensively evaluated regarding their photocatalytic effect before being applied on human skin, and that the formulation F11VGA undergoes significantly slower photo-oxidation than F11, it is possible to consider that the association of active antioxidant substances proposed to enhance skin conditions by preventing UV exposure damage, also protects skin from reactive oxygen species produced from TiO_2_ photocatalysis.

## 4. Studied Panel

It was recruited 45 volunteers, phototypes III to IV, aged 25–55 years. Informed consent was obtained from subjects before entering the study, which was approved by the Comitê de Ética em Pesquisa envolvendo Seres Humanos da Faculdade de Ciências Farmacêuticas de Ribeirão Preto—USP ethics committee.

### 4.1. Efficacy Evaluation

The multifunctional cosmetic formulations (F11VGA with and without UV filters) and the vehicle (F11 with and without UV filters) were randomly applied on the face and forearms of the volunteers, also a group of volunteer did not apply any formulation on a forearm (control group). The volunteers were guided to apply two formulations a day: the formulations with the sunscreens during the day and the formulations without the UV filters at night.

These studies formulations were applied during 90 days. The skin was evaluated after a single application (immediate effects, on the forearms) and after 30, 60 and 90 days application (log term effects, on face). The measurements on time points 30, 60 and 90 were made 24 h after the last application of the studied formulations, without any product on skin surface.

Clinical efficacy of the formulations was evaluated by biophysical techniques and skin image analyses according to the following parameters: stratum corneum water content, transepidermal water loss, skin color, and skin micro-relief. As well as skin images analysis techniques (digital photography imaging systems, 20 MHz ultrasound, and reflectance confocal microscopy) were applied to evaluate the efficacy of the studied formulations.

The stratum corneum water content was determined with a non-invasive, skin capacitance meter (Corneometer^®^ CM 825, Courage + Khazaka, Cologne, Germany), which measures capacitance and is entirely dependent on the water content in the skin. Different capacitance changes are converted into a digital measured value (arbitrary units) which is proportional to the skin humidity [38].

Barrier function was evaluated by measuring the transepidermal water loss (TEWL) (g/cm 2 h) using the Tewameter TM 300 (Courage & Khazaka). TEWL is considered an important measure of epidermal barrier function, a reduction on this parameter is linked with skin damage, inflammatory reactions and signs of aging [38,39].

Skin microrelief parameters were evaluated using a Visio Scan^®^ VC98 (Courage and Khazaka), which is a special high resolution UV-A light video camera developed especially to study the skin surface directly, and the Surface Evaluation of the Living Skin (SELS) method. The images show the structure of the skin and the level of dryness and the grey level distribution of the image is used to evaluate the following skin roughness parameters: skin roughness (Rt), skin smoothness (SEsm-proportional to width and form of the wrinkles) and number and width of the wrinkles (SEw) [40].

The Visioface^®^ digital photography imaging system (Courage and Khazaka) for evaluation of facial skin consist of a cabin attached to a high resolution digital camera (10 megapixels) and 200 white LED. This apparatus is connected to research software that enables evaluation of visible spots (with the color image), pores, wrinkles, and color differences in the target area, which is selected manually. The image is obtained with the aid of LED lights, which are strongly absorbed by the melanin present in the epidermis allowing for assessment of invisible spots that may become visible with skin aging and sun exposure [41].

The 20 MHz ultrasound Dermascan C^®^, (Cortex, Hadsund, Denmark) contains a transducer focus that is used for the attainment of two-dimensional transverse images, represented in the B-mode software. The ultrasonic wave (speed of 1,580 m/s) is partially reflected by the skin structure, giving rise to echoes of different amplitudes. To calculate the echogenicity, the number of pixels with low echogenicity is measured by means of the image analysis software and related to the total number of pixels [42].

*In vivo* reflectance confocal microscopy (RCM) is based on the imaging of light reflected by living tissue. The light source illuminates a small area of a three-dimensional sample, like the skin, and the illuminated region is then scanned into the detector through a small opening. The confocal images are registered in gray scale, where white represents the total reflected light and black is associated with the region without reflection. More light is reflected when the skin region contains structures with sizes similar to the wavelength of the light source systems as well as when the reflectance confocal microscopy is conducted by using a laser as light source. The images are recorded in the presence of an endogenous contrast, which can be provided by microstructures, such as melanin, or cellular organelles, such as hemoglobin [43].

### 4.2. Statistical Analysis

According to the sampling distribution of clinical evaluation data, the parametric Analysis of Variance was used to interpret the results using the software OriginPro 8 (OriginLab Corporation, Northampton, MA, USA).

## 5. Conclusions

In the assessment of the immediate effects of the studied formulations a moisturizing effect that lead to improved skin appearance lasting about 8 h was observed, requiring reapplication after this period so that the effect is maintained. Furthermore, the formulation containing the combination of active antioxidant substances under study was effective in improving the skin barrier function after a single application.

In the long-term study of clinical efficacy the studied formulation, with UV filters, vitamins and extracts under study, provided a significant improvement in skin barrier function and hydration into deeper layers of the epidermis, leading to improvement the skin's appearance by reducing wrinkles and roughness.

This study presents a multifunctional cosmetic formulation with established efficacy against signs of aging, assessed by advanced biophysical techniques and image analysis. This product can be suggested to preventing and improving skin conditions mainly related with photo aging. Also, this study presents the benefits of associating different active antioxidant substances in a single cosmetic formulation to skin. Still, this study presented innovative techniques that allow evaluating different parameters of the skin improving our knowledge about the skin aging process and enhancing the interpretation of efficacy evaluation of cosmetic formulations.
